# Gram differentiation of bacteria by acoustic-enhanced flow cytometry

**DOI:** 10.1099/jmm.0.002103

**Published:** 2026-02-19

**Authors:** Xiao Xuan Huang, Nadezda Urosevic, Timothy J. J. Inglis

**Affiliations:** 1 Department of Microbiology, PathWest Laboratory Medicine, QEII Medical Centre, Nedlands, WA, Australia; 2 School of Biomedical Sciences, The University of Western Australia, Nedlands, WA, Australia; 3 School of Medicine, The University of Western Australia, Nedlands, WA, Australia

**Keywords:** acoustic flow cytometry (AFC), blue laser channel 3-height (BL3-H), fluorescent dye SYTO 9, Gram differentiation of bacteria, Gram stain

## Abstract

**Introduction.** Recently, flow cytometry has gained the attention of clinical microbiologists for its ability to characterize bacterial species. This article shows how acoustic-enhanced flow cytometry, combined with the fluorescent dye SYTO 9, can differentiate between Gram-positive and Gram-negative bacteria.

**Gap Statement.** SYTO 9 is a cell membrane-permeable dye with a high affinity for nucleic acids in both living and non-living prokaryotic and eukaryotic cells and has been used as a counterstain to discriminate between live and dead cells in combination with other dyes. However, the consistency of its cell permeability in different bacterial species and its potential application to Gram differentiation has not been fully considered.

**Aim.** We sought to assess the suitability of the fluorescent dye, SYTO 9, to differentiate Gram-positive and Gram-negative bacteria by flow cytometry.

**Methodology.** A range of common Gram-positive and Gram-negative bacterial species were stained with SYTO 9, then processed using an acoustic-enhanced flow cytometer (Attune NxT, Thermo Fisher). The fluorescence emission data were gated and analysed in quadrant plots.

**Results.** Single and polymicrobial bacterial suspensions stained with SYTO 9 produced different fluorescence signals in Gram-positive and Gram-negative bacteria in the forward scatter-height/blue 3-height (FSC-H/BL3-H) quadrant. Gram-positive species (*Staphylococcus aureus*, *Enterococcus faecalis*, *Staphylococcus epidermidis*, *Streptococcus pneumoniae* and *Streptococcus pyogenes*) had higher fluorescence intensities in the BL3 channel than the Gram-negative species (*Escherichia coli*, *Klebsiella pneumoniae*, *Pseudomonas aeruginosa*, *Burkholderia thailandensis* and *Proteus vulgaris*) in both single and mixed cultures.

**Conclusion.** The FSC-H/BL3-H quadrant analysis of flow cytometer emission spectra from SYTO 9-stained bacterial suspensions segregated Gram-positive and Gram-negative bacteria into separate quadrants based on their different fluorescence intensities. This provides a single-dye flow cytometry method for Gram differentiation.

**Impact Statement**

Flow cytometer analysis of single-dye-stained bacteria has a range of potential applications in the clinical microbiology laboratory, where integration into specimen workflows would enable automation of a manual process, improving the time to completion of critical specimens and possibly increasing the sensitivity of detection of scanty bacterial cells.

## Introduction

The identification of clinically significant bacteria is a core component of clinical and veterinary microbiology. One of the earliest indications that bacteria are present in a clinical specimen is often seen during microscopic examination of a Gram-stained specimen, and even when automated laboratory equipment signals a positive culture, confirmation of its positive status relies on the Gram stain. Discovered by Hans Christian Gram in 1884 [1], Gram stain was modified for regular use in clinical microbiology [2]. Despite automation of the standard Gram stain method, the manual version followed by careful examination by light microscope remains a daily practice in clinical laboratories that process blood cultures and other critical specimens, such as spinal fluid, sputa and acute surgical wound swabs. The current manual Gram stain relies on a multi-stage process that includes an iodine–iodide mordant to stabilize the initial crystal violet primary stain before the final dilute carbol fuchsin counterstain [3]. The direct Gram stain method has remained in use for over 140 years because of its low cost, ease of use and key role in the initial stages of bacterial identification, despite limited understanding of the stain’s mechanism of action. The discovery that intrinsic features of bacterial cell walls were responsible for the differential staining of bacteria by Gram’s method came later [4,5].

Other methods of bacterial differentiation use different approaches and dyes for Gram differentiation. For example, Sizemore *et al.* [6] reported Gram-positive, but not Gram-negative, bacteria using fluorescein-labelled wheat-germ agglutinin examined by fluorescence microscopy. In contrast, Noda and Toel differentiated between Gram-positive and Gram-negative bacteria by staining cells with the anionic dye tetrabromophenolphthalein (TBP), followed by the cationic dye, octyl trimethylammonium (OTA), as reviewed by Popescu and Doyle [2]. This resulted in an inverted staining effect, where Gram-positive cells were not stained and Gram-negative cells stained positive with TBP/OTA [2]. In 1998, Mason *et al.* [7] combined two fluorescent dyes, hexidium iodide and SYTO 13, to distinguish Gram-positive from Gram-negative bacteria. Gram-positive bacteria retained both dyes and emitted red-orange fluorescence, while Gram-negative bacteria retained only SYTO 13 and thus emitted green fluorescence [7]. Despite these alternatives to Gram’s method, none have replaced the classical Gram stain in common clinical laboratory use.

We gained experience with SYTO 9-stained bacteria while investigating bacterial susceptibility to antimicrobial agents [8,9], where the stain was used to label bacterial blood culture isolates from cases of bloodstream infections, and then during development of a peptide nucleic acid fluorescence *in situ* hybridization (PNA-FISH) assay using acoustic-enhanced flow cytometry (AFC) [10]. During these studies, we used a wavelength range of 500–560 nm within the blue laser channel 1 (BL1) to capture maximum output emission but did not observe any differences in the emission spectra between various bacterial species in this wavelength range [8,10]. In a recent study, we used open-source data mining software (Orange, University of Ljubljana) for pattern recognition and as a prediction tool to identify differences in the emission spectra between Gram-positive and Gram-negative bacteria [11,12]. This software analysed cytometric patterns in all supplementary channels that were not essential according to the manufacturer’s manual, with an emphasis on variation between bacterial populations rather than on maximum emission. As a result, we discovered the most prominent and unforeseen variability between the emission spectra of Gram-positive and Gram-negative bacteria in the emission range of 655-735 nm of the blue laser channel 3 (BL3) that had not been identified previously by FlowJo [11,12]. Less prominent differences were seen in some other channels, of which violet laser channel 4 (VL4) was the second best [11,12]. In the present study, we applied the blue laser channel 3-height (BL3-H)/forward scatter-height (FSC-H) parameters to develop a method for bacterial Gram differentiation by FCM.

## Methods

### Bacterial isolates

Five Gram-positive bacterial species, namely *Staphylococcus aureus*, *Enterococcus faecalis*, *Staphylococcus epidermidis*, *Streptococcus pneumoniae* and *Streptococcus pyogenes,* and five Gram-negative bacterial species*, Escherichia coli*, *Klebsiella pneumoniae*, *Pseudomonas aeruginosa*, *Burkholderia thailandensis* and *Proteus vulgaris,* were used. The selection of bacterial species was made according to their clinical importance, except for *B. thailandensis*, which was chosen as an important outlier and as a near neighbour to *Burkholderia pseudomallei*, an endemic and clinically significant bacterial species in Western Australia that requires a higher level of laboratory containment. All bacterial species were from the American Type Culture Collection (ATCC) and were maintained by the Department of Microbiology, PathWest Laboratory Medicine (QEII Medical Centre, Nedlands, WA). Bacteria were resuscitated for analysis from bacterial stocks onto 5% horse blood agar, streaked out to obtain single-colony growth and incubated at 37 °C overnight. A single colony was then picked, inoculated in 10 ml Tryptic Soy Broth (TSB) and incubated at 37 °C overnight. These cultures were briefly resuscitated the next day and used for flow cytometer analysis, as described below.

### Bacterial culture preparation for FCM

On the day of FCM, overnight bacterial cultures were resuscitated in a 1-in-10 vol. of fresh media for 1 h, followed by staining with SYTO 9. Prior to staining, a 1 ml aliquot of resuscitated bacterial culture was transferred into a 1.5 ml microcentrifuge tube and centrifuged at 10,000***g*** for 5 min. After aspirating the supernatant, the bacterial pellet was resuspended in 1 ml of 0.1 μm-filtered Hank’s Balanced Salt Solution (HBSS). The sample was centrifuged one more time at 10,000***g*** for 5 min, followed by resuspension in 1 ml of HBSS. Samples were stained with 1 µl SYTO 9 for either 1 or 5 min, respectively, and analysed on the flow cytometer. Bacterial concentrations in single-species suspensions were quantified by the flow cytometer, with final concentrations ranging from 10^5^ to 10^7^ c.f.u. ml^−1^. The limit of bacterial detection was determined as 10^3^–10^4^ c.f.u. ml^−1^, as reported previously [10].

### Preparation of polymicrobial bacterial suspensions

Prior to preparation of polymicrobial suspensions, 100 ml of overnight bacterial cultures grown in TSB was diluted with additional 900 ml of TSB and incubated for 1 h at 37 °C to resuscitate bacteria. This was followed by two washes with HBSS. The bacterial concentrations in resuscitated bacterial cultures were determined using FCM of SYTO 9-stained cells and adjusted to the desired concentrations. For preparing polymicrobial suspensions, one Gram-positive and one Gram-negative bacterial species were mixed in either equal proportions at 1 : 1 (5.00×10^5^ c.f.u. ml^−1^ for each bacterial species) or unequal proportions 1 : 10, containing 5.00×10^5^ c.f.u. ml^−1^ of Gram-positive and 5.00×10^6^ c.f.u. ml^−1^ of Gram-negative bacteria, respectively, and stained with SYTO 9 for 1 min before being analysed on the flow cytometer.

### Exposure of bacterial suspensions to 70% ethanol

To test whether the cell wall damage would affect segregation of Gram-positive from Gram-negative bacteria in the quadrant analysis, the bacterial pellets were resuspended in 1 ml of 70% ethanol (v/v) for 5 min at room temperature after two washes in HBSS. The samples were then centrifuged at 10,000***g*** for 5 min, followed by resuspension in 1 ml HBSS. The samples were stained using 1 µl SYTO 9 for 1 min and analysed on the flow cytometer.

### Flow cytometry

An acoustic flow cytometer (Attune NxT, Cellular Analysis, Thermo Fisher, Oregon) was used to analyse all bacterial samples. The flow cytometer was configured with four excitation lasers: blue (488 nm), violet (405 nm), yellow (561 nm) and red (637 nm), and with 16 detection channels for a broad detection range. System initialization and performance tests were conducted prior to each experiment to optimize performance. To avoid bacterial carry-over, 1 ml of 10% bleach, followed by 1 ml of 0.1 µm-filtered Milli-Q water, was passed through the sampling port (sipper) after each sample.

## Results

### FCM set up and quadrant gating

**Fig. 1.**
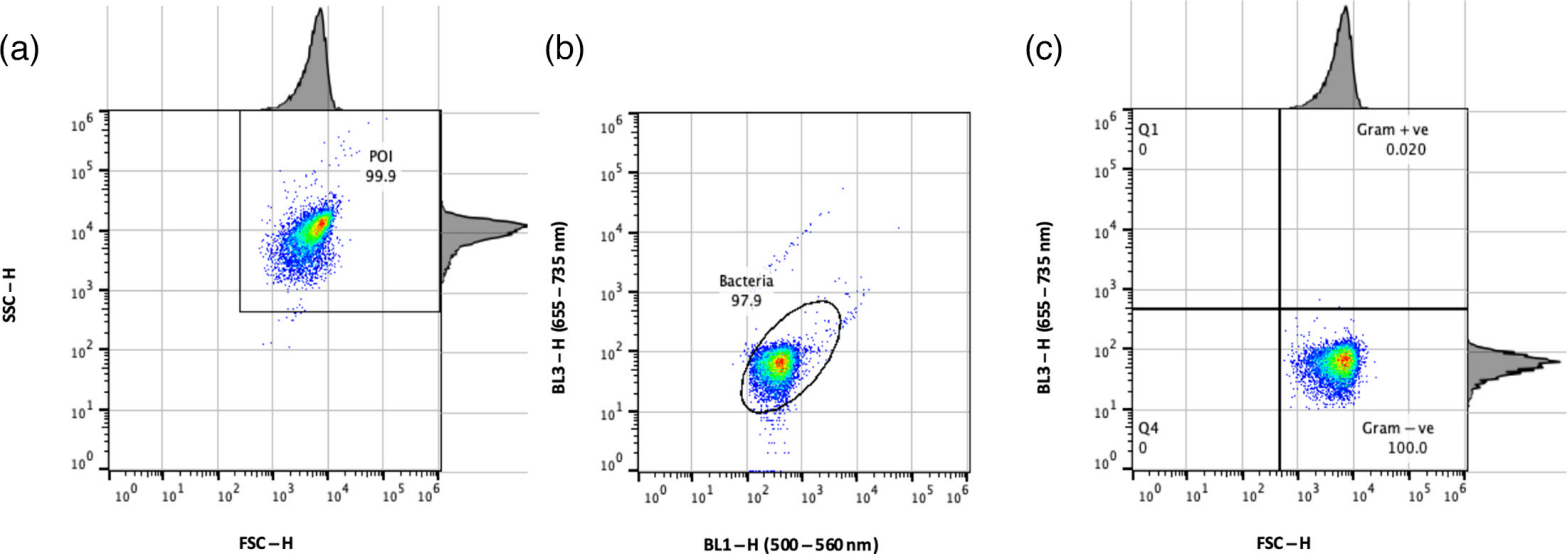
Gating procedure for Gram differentiation in pure cultures of *P. vulgaris* stained with SYTO 9. POI was identified based on FSC-H/SSC-H signals (**a**). The bacterial population was identified based on SYTO 9-positive signals in the BL1-H/BL3-H channels (**b**). Last, the bacterial population was plotted based on the relative size of bacterial cells (FSC-H) and SYTO 9 signal intensity (BL3-H) using quadrant gate analysis to differentiate Gram-positive (Gram +ve) from Gram-negative (Gram -ve) bacteria (**c**). The position of the bacterial gate in (b) was dependent on the positive signals in the BL1 and BL3 channels, which were species specific, while the quadrant gates were constant and were not affected by species specificity. SSC-H, side scatter-height.

Having demonstrated the value of bacterial analysis for bacterial differentiation on the BL3 channel previously [11,12], a gating method was developed to include BL3-H in addition to BL1-H. Briefly, FSC and side scatter (SSC) were first set up on the bivariate scatter plot using logarithmic scales (Fig. 1a). A population of interest (POI) was gated using BL1-H (500–560 nm) and BL3-H (655–735 nm), respectively, and labelled as ‘bacteria’ (Fig. 1b). The position of the bacterial population in the quadrant plot assigned by parameters FSC-H and BL3-H (655–735 nm) (Fig. 1c) was subsequently used for determination of Gram status.

### Optimization of staining with SYTO 9

**Fig. 2.**
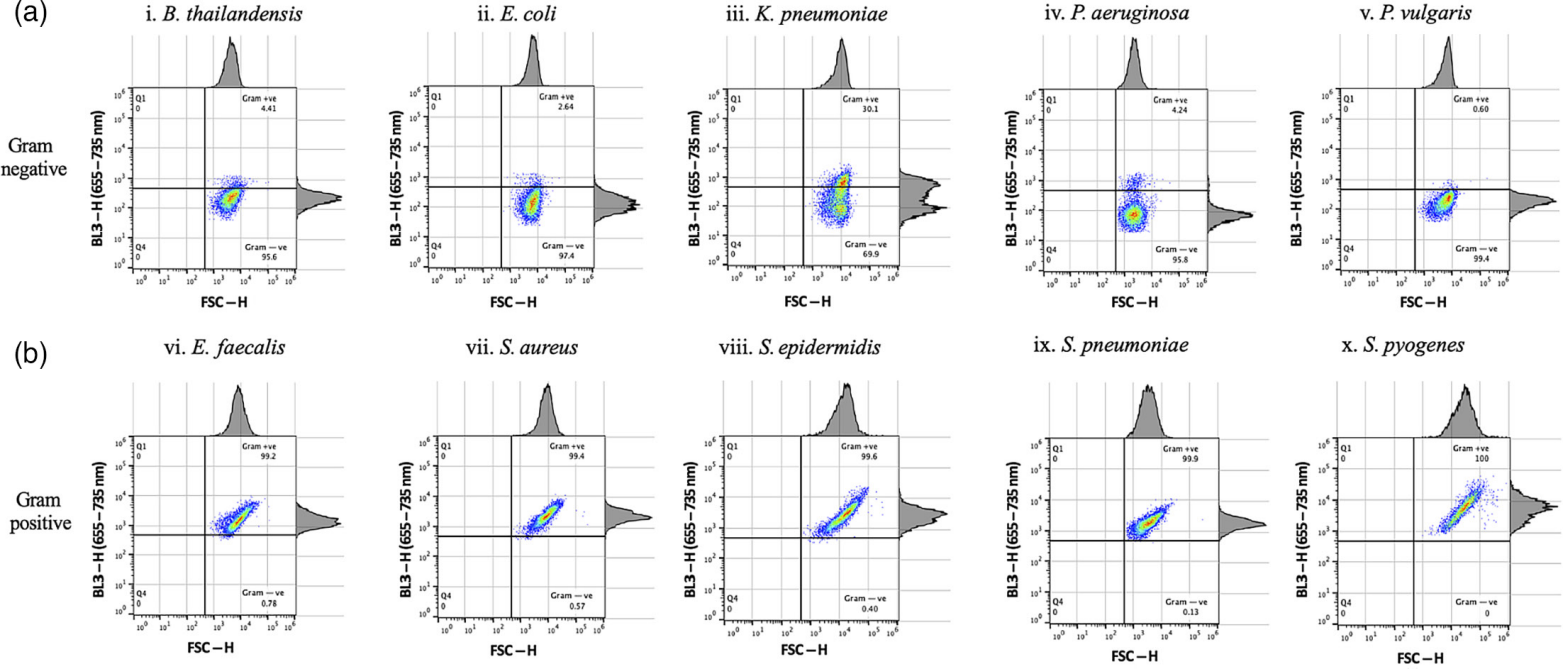
Gram differentiation of single overnight bacterial cultures stained with SYTO 9 for 5 min. Five different species of Gram-negative bacteria, namely *B. thailandensis, E. coli, K. pneumoniae, P. aeruginosa* and *P. vulgaris* [a(i–v)], and five Gram-positive bacterial species, *E. faecalis, S. aureus, S. epidermidis, S. pneumoniae* and *S. pyogenes* [b(i–v)], were analysed using FSC-H/BL3-H quadrant analysis following SYTO 9 staining. A segregation between Gram-negative bacteria (bottom right quadrant) and Gram-positive bacteria (top right quadrant) was observed, with some deviations for *K. pneumoniae* [a(iii)] and *P. aeruginosa* [a(iv)] shown. The experiment was repeated twice.

**Fig. 3.**
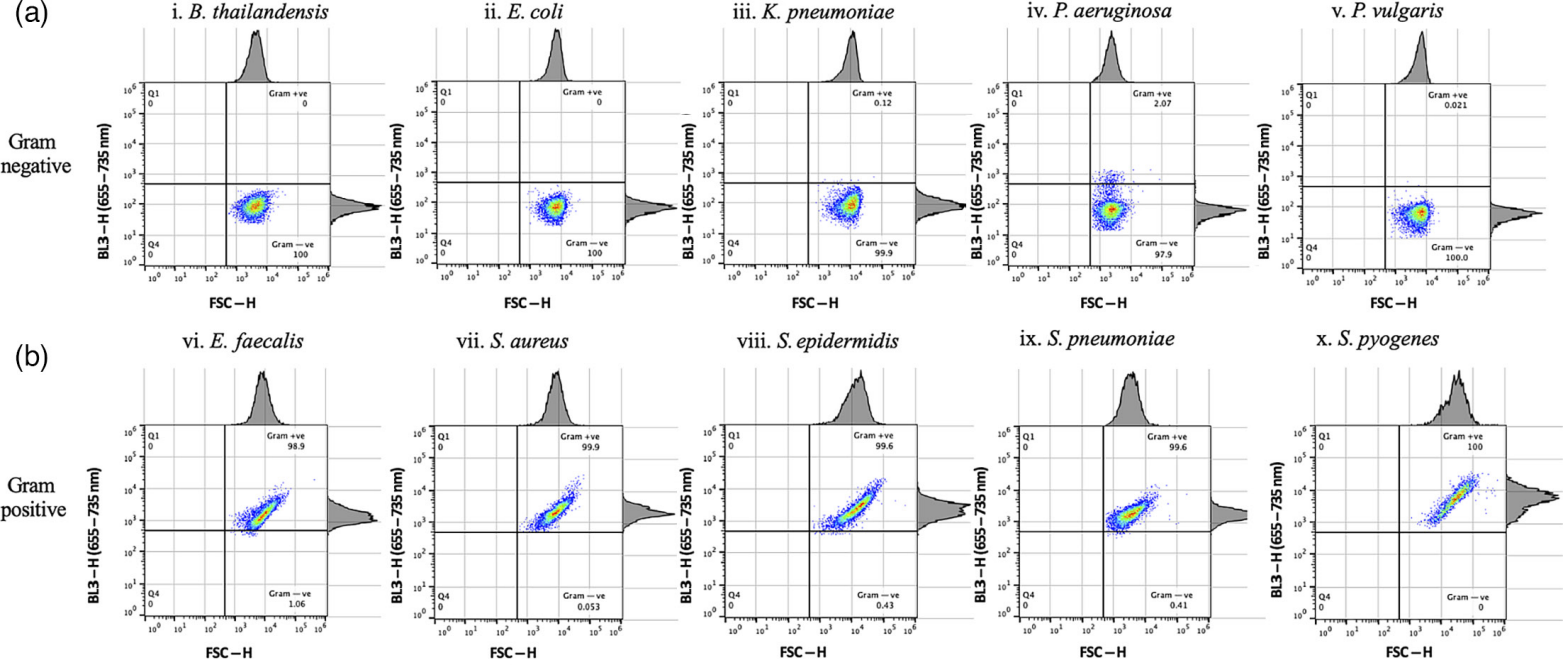
Gram differentiation of single overnight bacterial cultures following 1 min of SYTO 9 staining. An improved segregation between Gram-negative bacteria [a(i–v)] and Gram-positive bacteria [b(i–v)] is shown in the bottom and top right quadrants, respectively. A slight deviation for *P. aeruginosa* [a(iv)] is observed, while all Gram-positive bacteria were in the correct quadrants. The experiment was repeated twice.

The effect of 5 min (Fig. 2) and 1 min SYTO 9 staining (Fig. 3) prior to flow cytometer analysis was assessed to optimize separation of Gram-positive and Gram-negative bacterial populations in quadrant plots. Populations of five Gram-positive and five Gram-negative bacterial species produced distinct BL3-H emission intensity patterns in the corresponding quadrant plots (Figs2  3). Populations of all five Gram-negative bacterial species had low fluorescence intensities in the BL3-H channel and were captured in the lower right quadrant (Figs2a  3a). In contrast, all Gram-positive species emitted higher fluorescence in the BL3-H channel, appearing in the upper right quadrant (Figs2b  3b). Stark differences between 5 and 1 min staining durations were observed in the BL3-H channel for Gram-negative bacteria. Most notably, *K. pneumoniae* spilled into the Gram-positive domain when stained for 5 min [Fig. 2a(iii)]. The four other Gram-negative species also produced higher BL3-H signals after 5 min of staining. A subpopulation of *P. aeruginosa* crossed into the area occupied by Gram-positive bacteria when stained for either 5 or 1 min [Figs2a(iv)  3a(iv), respectively]. Despite these areas of overlap, the majority of each bacterial population was located in the defined upper or lower quadrant domains that corresponded to their Gram properties, especially following 1 min staining. These experiments were repeated several times, especially for *K. pneumoniae,* until satisfactory quadrant separation was achieved using bacterial cultures in the growth phase and with a shorter staining time of 1 min (Fig. 3). All subsequent experiments were therefore carried out with resuscitated cultures at the 1 min SYTO 9 staining duration.

### Separation of Gram-positive and Gram-negative bacteria in mixed cultures

**Fig. 4.**
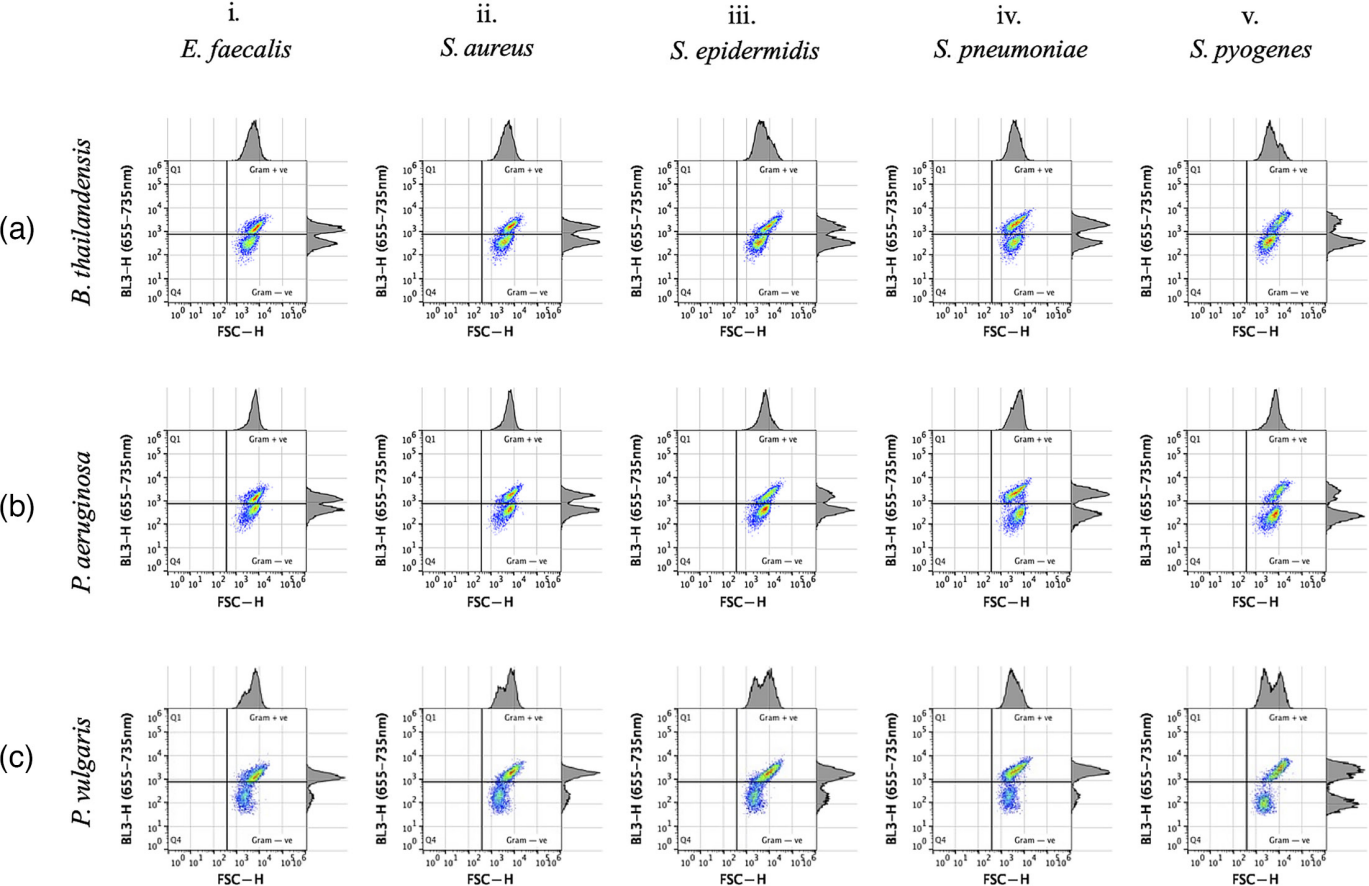
Gram differentiation of polymicrobial bacterial cultures stained with SYTO 9. Equal volumes of one of five Gram-positive (**i–v**) and one of three Gram-negative (**a–c**) bacterial species were mixed following overnight growth and prior to staining with SYTO 9. Quadrant analysis indicated a clear segregation between all five Gram-positive and three Gram-negative bacterial species, *B. thailandensis* [a(i–v)], *P. aeruginosa* [b(i–v)] and *P. vulgaris* [c(i–v)], in mixed cultures. The experiment was repeated twice.

When equal parts of a Gram-positive and a Gram-negative bacterial suspension were combined to test the efficacy of bacterial cell population bivariate quadrant analysis in mixed cultures, two distinct populations could be easily identified within upper and lower quadrant domains in suspensions containing Gram-negative bacteria, *B. thailandensis, P. aeruginosa* or *P. vulgaris,* mixed with different Gram-positive bacteria (Fig. 4a–c, respectively).

**Fig. 5.**
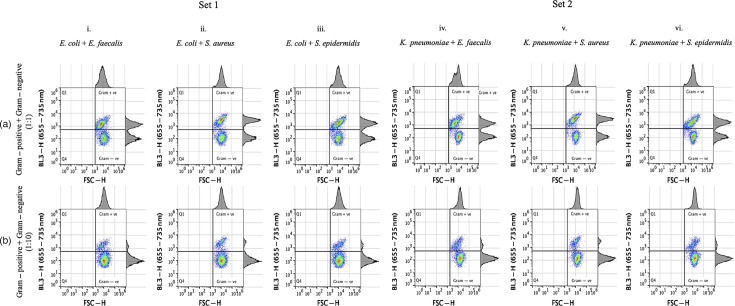
Limit of bacterial detection and Gram differentiation in mixed bacterial cultures containing one Gram-negative and one Gram-positive bacterial species. Two sets of mixed bacterial suspensions were prepared: Set 1, containing *E. coli* mixed with either *E. faecalis* (**i**), *S. aureus* (ii) or *S. epidermidis* (iii), and Set 2, containing *K. pneumoniae* mixed with either *E. faecalis* (iv), *S. aureus* (v) or *S. epidermidis* (**vi**). The mixed cultures were prepared in a 1 : 1 ratio (**a**) and a 1 : 10 ratio, having ten times more Gram-negative than Gram-positive bacterial species (**b**). All bacterial species were adjusted to 5.00x10^5^ or 5.00x10^6^ c.f.u. ml^−1^, respectively, prior to mixing. This experiment was performed once.

Bacterial suspensions were also prepared to contain 1 : 1 or 1 : 10 of Gram-positive (*E. faecalis, S. aureus* or *S. epidermidis*) to Gram-negative bacteria (*E. coli* and *K. pneumoniae*), respectively. Following FCM and quadrant analysis, a clear separation of Gram-positive from Gram-negative bacteria was observed across all samples (Fig. 5). While the ten-fold difference in the concentration of Gram-positive to Gram-negative organisms did not affect detection of distinct bacterial cell populations, it generated a proportionally smaller population of non-dominant Gram-type bacteria in the quadrant plots (Fig. 5b). At a 100-fold difference, only the dominant Gram-type bacteria were observed, indistinguishable from single-species suspensions, supporting the ten-fold predominance limitation of this procedure for mixed suspensions of Gram-positive and Gram-negative bacteria.

### Effect of cell wall integrity on fluorescence signal intensity in quadrant analysis

**Fig. 6.**
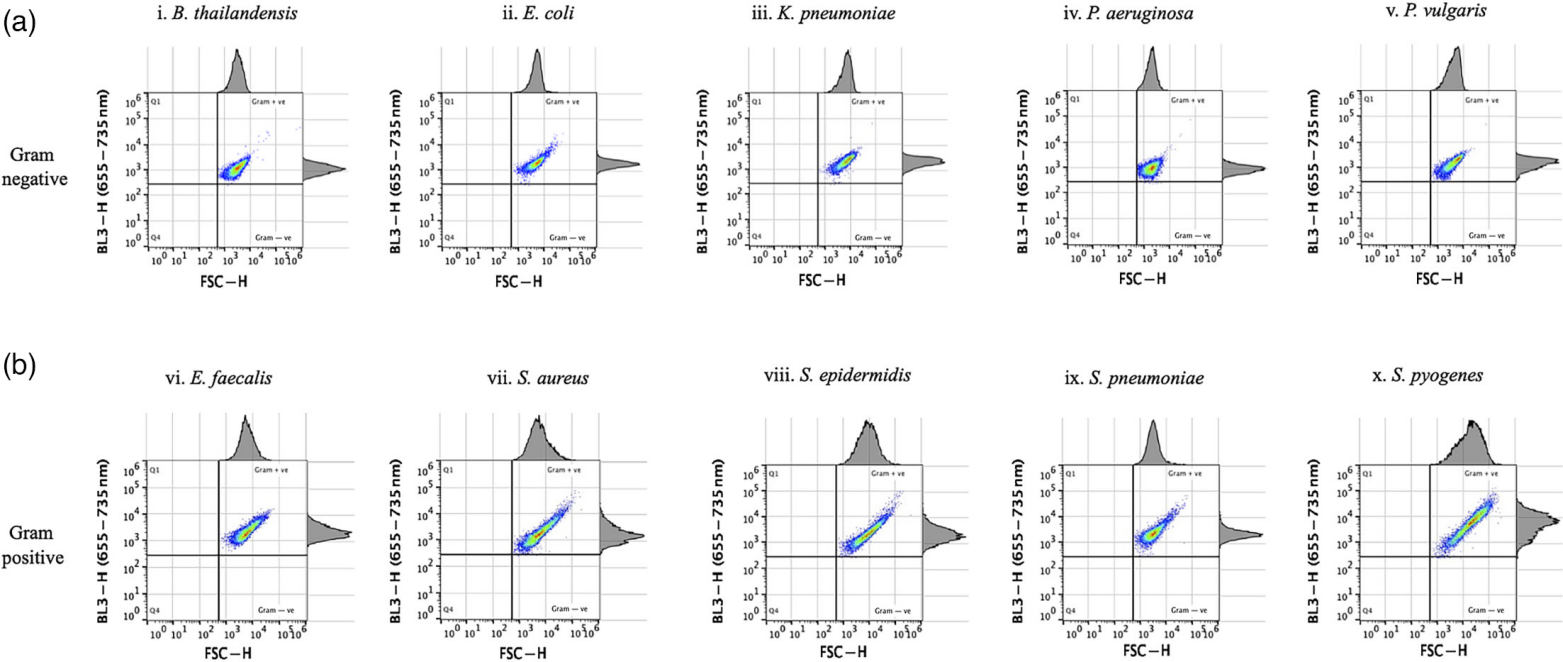
Effect of the cell wall integrity on the Gram differentiation by quadrant analysis. All Gram-negative (**a**) and Gram-positive (**b**) bacterial species have been treated with 70% ethanol (v/v) for 5 min prior to 1 min staining with SYTO 9. As shown in row (a), all Gram-negative bacteria treated with alcohol demonstrated more intense fluorescence in the BL3 channel and segregated to the top right quadrant specific for the Gram-positive bacteria (**b**). The experiment was repeated twice.

The SYTO 9 fluorescent dye is known to equally penetrate both live and dead bacterial cells. We exposed bacteria to 70% ethanol for 5 min at room temperature prior to SYTO 9 staining. The shape and positions of bacterial populations after treatment with alcohol changed significantly, as observed in quadrant plots (Fig. 6). A prominent cell population shift occurred in which Gram-negative organisms changed from the lower to the upper quadrant (Fig. 6a), while the Gram-positive populations following alcohol treatment remained in the same quadrant, with only discrete changes not visible to the unaided eye (Fig. 6b).

## Discussion

Limitations of the conventional Gram stain method, such as the poorly controlled duration of the decolorization step and subjective interpretation of results, have prompted the development of more objective approaches to Gram differentiation. Advances in flow cytometer analysis of eukaryotic cells, the proliferation of fluorescent dyes for specific intra- and extracellular components, and the development of sophisticated instruments have all contributed to microbial FCM progress, including efforts to achieve Gram differentiation [6,7, 13, 14]. Differential staining of Gram-positive and Gram-negative bacteria was previously performed with a variety of compounds and fluorophores that either had discrepant affinity for bacterial cell components, opposing cell membrane-permeant properties or divergent decolourization of Gram-positive and Gram-negative bacteria [6,7, 14, 15]. All these previous methods relied on multiple fluorophores or multiple steps to achieve bacterial cell Gram-type differentiation. Here, we present a simple, one-step, FCM-based procedure that uses a single fluorescent dye, SYTO 9, to discriminate between Gram-positive and G bacteria with different signal intensities in the BL3-H channel.

 Despite the fact that SYTO 9 has been successfully used as a counterstain in microscopic testing of live/dead bacteria for many years, FCM has pointed to anomalies in staining intensities among different bacterial species [16,18]. These anomalies have somewhat confounded the accuracy of cell viability and enumeration tests [16,19]. However, in the approach described here, species-specific variations have not significantly influenced the overall accuracy of Gram differentiation of live bacterial cell populations in liquid suspension. Perceived limitations of the current procedure are cell membrane integrity requiring an additional hour of bacterial resuscitation in fresh media, and the minimum bacterial concentration at and above 10^3^–10^4^ c.f.u. ml^−1^ to discriminate individual cells from the background noise. The exquisite sensitivity of single-cell analysis by the acoustic flow cytometer enables an accurate estimate of cell numbers in the BL1 channel [10] as well as Gram status in the BL3 channel, as described here. This could also serve as a preliminary step prior to automated antimicrobial susceptibility testing [8,9], which might be advantageous over similar platforms, including the Sysmex UF-5000. Moreover, despite some similarities between our procedure and the Sysmex UF-5000, such as blue-laser excitation, acoustic cell focusing of the Attune has been designed more recently for higher sensitivity than the hydrodynamic focusing [8] used by Sysmex. In addition, the emitted signals in these two systems are analysed and presented differently. While the Gram status of bacterial populations in urine was predicted from an angle between FSC and fluorescent light height by the Sysmex UF-5000 [20], in our model, the size and intensity of the signal in the BL3-H channel determine the position of individual cells in the quadrant plots, providing an unequivocal cut-off between Gram-positive and Gram-negative organisms, especially in mixed cultures subject to the ten-fold bacterial predominance limitation. All of these might suggest an advantage of our procedure over Sysmex; however, a definitive answer cannot be provided until these two systems have been evaluated in parallel.

When compared with traditional Gram stain, our method is more precise in sample handling and less biassed in result interpretation. Sample pre-processing time is ~20 min, allowing for simultaneous preparation of multiple samples. The advantage over traditional Gram staining for clinical specimens lies in its speed, impartiality in result recording and the likelihood of earlier detection of bacteria at lower concentrations, with instantaneous delivery of quantitative and qualitative characteristics of the bacteria in the specimen.

In conclusion, we applied acoustic FCM for Gram differentiation of bacterial cells. This was achieved using a single fluorescent dye, SYTO 9, analysed by an acoustic flow cytometer on its blue laser-height channel. When flow cytometer data were displayed in bivariant quadrant analysis, suspensions of five medically important Gram-positive and five Gram-negative bacterial species were easily separable into different quadrants based on their Gram properties, regardless of whether they were part of single-species or mixed cultures. These findings provide a foundation for future application of this method to the detection and initial Gram classification of common bacteria in liquid cultures, such as blood culture fluid and other specimen types.

## Abbreviations

ATCC: American Type Culture Collection
BL3-H: blue laser channel 3-height
FCM: flow cytometry
FSC-H: forward scatter-height
HBSS: Hank’s Balanced Salt Solution
OTA: octyl trimethylammonium
PNA-FISH: peptide nucleic acid fluorescence *in situ* hybridization
POI: population of interest
TBP: tetrabromophenolphthalein
TSB: tryptic soy broth
